# Possible molecular mechanisms of persistent pollen tube growth without *de novo* transcription

**DOI:** 10.3389/fpls.2022.1020306

**Published:** 2022-11-24

**Authors:** Kazuki Motomura, Naoya Sugi, Atsushi Takeda, Shohei Yamaoka, Daisuke Maruyama

**Affiliations:** ^1^ College of Life Sciences, Ritsumeikan University, Kusatsu, Japan; ^2^ Japanese Science and Technology Agency, PRESTO, Kawaguchi, Japan; ^3^ Institute of Transformative Bio-Molecules, Nagoya University, Nagoya, Japan; ^4^ Kihara Institute for Biological Research, Yokohama City University, Yokohama, Japan; ^5^ Graduate School of Biostudies, Kyoto University, Kyoto, Japan

**Keywords:** pollen tube growth, pollen tube guidance, male germ unit, vegetative nucleus, plant reproduction, gene expression

## Abstract

The vegetative cell nucleus proceeds ahead of a pair of sperm cells located beneath the pollen tube tip during germination. The tip-localized vegetative nucleus had been considered to play a pivotal role in the control of directional pollen tube growth and double fertilization. However, we recently reported the female-targeting behavior of pollen tubes from mutant plants, of which the vegetative nucleus and sperm nuclei were artificially immotile. We showed that the apical region of the mutant pollen tubes became physiologically enucleated after the first callose plug formation, indicating the autonomously growing nature of pollen tubes without the vegetative nucleus and sperm cells. Thus, in this study, we further analyzed another *Arabidopsis thaliana* mutant producing physiologically enucleated pollen tubes and discussed the mechanism by which a pollen tube can grow without *de novo* transcription from the vegetative nucleus. We propose several possible molecular mechanisms for persistent pollen tube growth, such as the contribution of transcripts before and immediately after germination and the use of persistent transcripts, which may be important for a competitive race among pollen tubes.

## Introduction

A pollen is a small male gametophyte produced by flowering plants. *Arabidopsis thaliana* produces tricellular pollen grains consisting of vegetative cells that contain an eyeglasses-shaped pair of sperm cells. The two sperm cells are enclosed by the inner vegetative plasma membrane (IVPM), an endomembrane that originates from the plasma membrane of the host vegetative cell. This reflects the phagocytic internalization of the sperm cell precursor during male gametogenesis. In addition to the symbiotic feature, the male germ unit (MGU) is formed by a complex of vegetative nuclei and sperm cells, representing the uniqueness of pollen cell biology (Mogensen, 1992). After pollen tube germination, the MGU is localized in the apical area approximately 50–100 µm away from the tip of the pollen tube and maintains the triplet structure, keeping the vegetative nucleus ahead of the sperm cells. The directional growth of the pollen tube in response to various guidance cues in the pistil is a complex process. Regardless, the apical MGU localization ensures precise sperm cell delivery before its double fertilization by rupturing the tip of the pollen tube inside the ovule (Hamamura et al., 2011; McCue et al., 2011; Denninger et al., 2014; Higashiyama and Takeuchi, 2015).

Although the molecular mechanisms of the apical MGU transport are poorly understood, available genetic evidence supports that the vegetative nucleus and sperm cells in the MGU have their respective motive forces in the pollen tube. For example, the linker of the nucleoskeleton and cytoskeleton (LINC) complex plays a key role in vegetative nucleus transport. This nuclear envelope complex regulates nucleus morphology and migration in various plant cells *via* mechanical force transmission, which is generated by motor proteins and cytoskeletons in the cytosol (Tatout et al., 2014). Zhou and Meier (2014) reported that in *A. thaliana*, simultaneous loss of the functionally redundant LINC complex proteins, WPP domain-interacting tail-anchored protein 1 (WIT1) and WIT2, on the outer nuclear envelopes causes the passive migration of the vegetative nucleus dragged by the sperm cells (Zhou and Meier, 2014). In a parallel study, we observed over-accumulation of callose (β-1,3-glucan) at the cell wall of sperm cells expressing the gain-of-function mutant protein, CALLOSE SYNTHASE 3 (*cals3m*), fused with the sperm cell-specific *HTR10* promoter (*pHTR10:cals3m*) (Motomura et al., 2021). These abnormal sperm cells are usually observed at the basal region of the pollen tube, probably due to the loss of sperm cell motility (Motomura et al., 2021).

The decreased motilities of the vegetative nucleus and the sperm cells observed in the *wit1 wit2* double mutant and *pHTR10:cals3m* (hereafter referred to as *SC-cal*)-expressing *Arabidopsis* plants enabled us to better understand the hidden potential of the pollen tube to grow autonomously irrespective of the MGU. Interestingly, the *wit1 wit2* mutant expressing *SC-cal* produced pollen tubes with the vegetative nucleus and sperm nuclei located behind the callose plugs in its basal region (Motomura et al., 2021). Callose plugs are callose-rich cell wall partitions specifically found in pollen tubes, where phasing formation maintains most of the protoplasm at its apical region (Franklin-Tong, 1999). The first callose plug formation isolates immotile MGUs, leading to physiologically anucleated growth of the pollen tubes in *wit1 wit2 SC-cal* plants within 3 h after germination. Surprisingly, *wit1 wit2 SC-cal* pollen tubes not only displayed continuous growth but also responded to pollen tube-attractant signals and reached the ovule. These results suggest that the pollen tube is a robust stand-alone system that can work even without the *de novo* transcript supply from the MGU (Motomura et al., 2021).

*DROP1* and *DROP2*, also known as *LRL1* and *LRL2*, respectively, are members of subfamily XI of the basic helix-loop-helix (bHLH) transcription factors. The *drop1 drop2* double mutant showed loss of sperm cells in pollen grains, exhibiting normal directional growth toward attractant peptides and pollen tube discharge in the ovule (Zhang et al., 2017). This mutant is an excellent material to demonstrate the irrelevance of sperm cells in various pollen tube behaviors, elucidating the significance of the vegetative nucleus. A similar sperm cell-absent phenotype was also induced by double mutations in *BONOBO1* (*BNB1*) and *BNB2*, which encode subfamily VIIIa bHLH proteins that are putative partners of DROP1 and DROP2 (Yamaoka et al., 2018). In the present study, we further investigated whether *wit1 wit2* double mutations abolished the apical transport of the vegetative nucleus in the *bnb1 bnb2* double mutant. We show that most sperm cell-absent pollen tubes carrying *bnb1 bnb2 wit1 wit2* quadruple mutations retain the vegetative nucleus in their basal regions. Importantly, these pollen tubes maintain the capabilities of tip-growth and attraction to an ovule after isolation of the vegetative nucleus despite their anucleated condition, supporting the stand-alone behavior of the *wit1 wit2 SC-cal* pollen tubes (Motomura et al., 2021). Based on these results, we discuss when and how pollen tubes obtain their versatile abilities concerning *de novo* gene expression in the vegetative nucleus.

## Materials and methods

### Plant material and growth conditions

*A. thaliana* Columbia-0 (Col-0) was used as the wildtype (WT) plant and the background of all the mutants. The *pRPS5A:H2B-tdTomato* plasmid was provided by Dr. Kurihara (Maruyama et al., 2013). The *wit1-1 wit2-1* double mutant was kindly provided by Dr. Meier and Dr. Tamura (Zhou and Meier, 2014). The *bnb1* (GK-277A11) and *bnb2* (SALK_031573) mutants have been previously described (Yamaoka et al., 2018). Plants were grown in soil at 22°C under continuous light conditions. *Agrobacterium*-mediated plant transformation was performed by the floral dipping method using *Agrobacterium* strain GV3101 (Clough and Bent, 1998).

### Pollen grain analysis

Mature pollen grains from opened flowers were mounted on a 5% sucrose solution for further imaging analysis. Differential interference contrast (DIC) and fluorescence images were acquired using a confocal laser-scanning microscope (Leica TCS SP8, Wetzlar, Germany).

### Pollen tube analyses

For the *in-vitro* pollen tube growth assay, pollen grains were incubated on pollen germination medium (0.01% boric acid, 5 mM CaCl_2_, 5 mM KCl, 1 mM MgSO_4_, 10% sucrose, adjusted pH to 7.5 with 1 N KOH, and 1.5% NuSieve GTG agarose) supplemented with 10 μM epibrassinolide (Muro et al., 2018). Pollen tubes 3 h after germination (HAG) were observed using an Axio Imager M1 (Zeiss, Jera, Germany). For the *in-vivo* single pollen tube guidance assay, a single pollen grain was picked up by an eyelash under an MVX10 macro zoom microscope (Olympus, Tokyo, Japan) and attached to the stigma of a WT pistil, as previously described (Zhang et al., 2017). In the case of the quadruple mutant pollen, teardrop-shaped pollen grains were selected from pollen from the +/*bnb1 bnb2 wit1 wit2* mutant. The pistils one day after pollination were dissected in aniline blue solution (0.1% [w/v] aniline blue, 0.1 M K_3_PO_4_) and observed using the Axio Imager M1.

## Results

**Figure 1:**
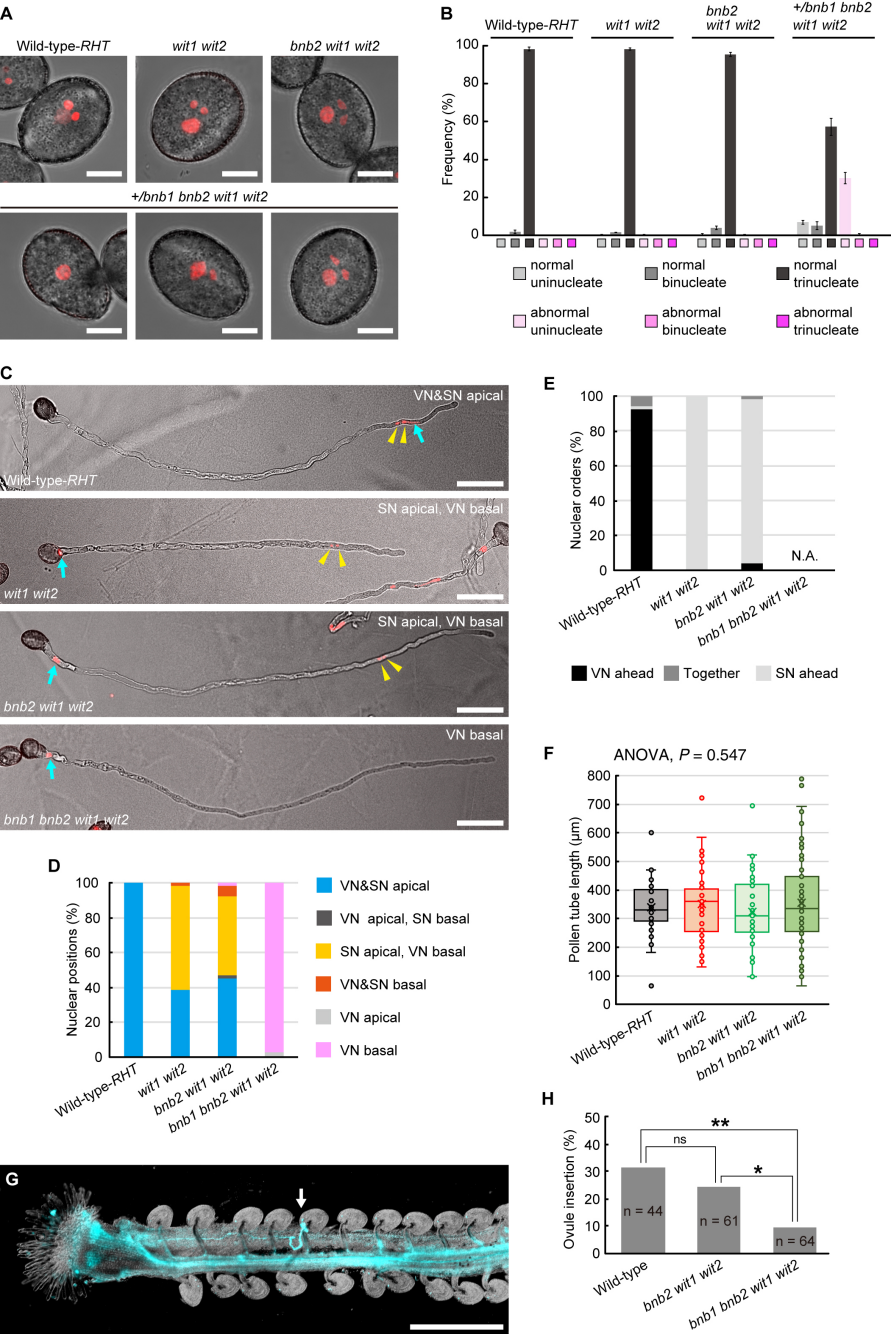
Pollen tube behavior in +/*bnb1 bnb2 wit1 wit2* mutant plants. **(A)** Representative images of mature transgenic pollen carrying the *pRPS5A:H2B-tdTomato* (*RHT*) plasmid of the wildtype (WT) Col-0 plant (WT-*RHT*), *wit1 wit2* double homozygous mutant, *bnb2 wit1 wit2* triple homozygous mutant or +/*bnb1* heterozygous, and *bnb2 wit1 wit2* triple homozygous mutant (+/*bnb1 bnb2 wit1 wit2*). The three images represent an abnormal uninucleate (left), a normal binucleate (center), and a normal trinucleate (right) pollen from the +/*bnb1 bnb2 wit1 wit2* mutant. Scale bars: 10 μm. **(B)** Pollen nucleation frequencies in the four genotypes observed in **(A)**, considering both the number of nuclei and shape of pollen grains. Normal indicates pollen of normal contour. At least three independent observations were conducted, with a total of 572 to 1,160 pollen grains. **(C)** Representative images of WT-*RHT*, *wit1 wit2*, *bnb2 wit1 wit2*, and *bnb1 bnb2 wit1 wit2* pollen tubes 3 h post-germination *in vitro*. Blue arrows and yellow arrowheads indicate vegetative nuclei (VN) and sperm nuclei (SN) pairs, respectively. Scale bars: 50 μm. **(D, E)** Nuclear positions **(D)** and nuclear orders **(E)** analyzed in the WT-*RHT*, *wit1 wit2*, *bnb2 wit1 wit2*, and *bnb1 bnb2 wit1 wit2* mutant pollen tubes. For each plant genotype, 51 to 65 pollen tubes were observed. **(F)** Box-and-whisker plots of pollen tube length analyzed in the same samples as in **(D)** and **(E)**. Note that we observed only *bnb1 bnb2 wit1 wit2* quadruple pollen tubes with one nucleus out of 65 pollen grains from +/*bnb1 bnb2 wit1 wit2* mutants in **(C)** to **(F)**. **(G)** Representative image of an ovule penetrated by a *bnb1 bnb2 wit1 wit2* quadruple mutant pollen tube (white arrow). In the *in-vivo* single-pollen-tube guidance assay, a pistil was harvested one day after pollination and observed after aniline blue staining. Scale bar: 500 μm. **(H)** Ovule-insertion analyzed in the *in-vivo* single pollen tube guidance assay of the WT, *bnb2 wit1 wit2*, and *bnb1 bnb2 wit1 wit2* mutant pollen. Statistics: Chi-square test with a *p*-value adjustment by the Hochberg algorithm. ns, not significant. *P = 0.0459. **P = 0.0095.

To examine the motility of the vegetative nucleus in the pollen tubes of the *bnb1 bnb2 wit1 wit2* quadruple mutant, we generated an *A. thaliana* mutant that was heterozygous for *bnb1* (+/*bnb1*) and homozygous for *bnb2*, *wit1*, and *wit2* since *bnb1 bnb2* double homozygous plants cannot be recovered due to severe male lethality (Yamaoka et al., 2018). All mutant and WT plants were transformed with the *pRPS5A:H2B-tdTomato* (*RHT*) ubiquitous nuclear marker gene (Maruyama et al., 2013). The results showed that most pollen grains had tricellular MGUs containing two condensed sperm nuclei and a relatively dispersed single vegetative nucleus in the WT-*RHT*, *wit1 wit2* double mutant, and *bnb2 wit1 wit2* triple mutant (**Figures 1A,B**). However, in the +/*bnb1 bnb2 wit1 wit2* mutant, only approximately 60% of the pollen was trinucleate, while the remaining pollen showed aberrant uninucleate or binucleate cells (**Figures 1A,B**). Notably, unicellular pollen contained a single vegetative nucleus and usually showed abnormal teardrop-shaped morphology with tiny protrusions due to the abortion of the male germ cells. These pollen phenotypes were comparable to those observed in a previous study with +/*bnb1 bnb2* plants (Yamaoka et al., 2018), suggesting little or no effect on *wit1 wit2* double mutations during pollen formation. The +/*bnb1 bnb2 wit1 wit2* mutant should produce *bnb2 wit1 wit2* triple mutant pollen and *bnb1 bnb2 wit1 wit2* quadruple mutant pollen in a 1:1 ratio. Thus, we concluded that aberrant unicellular or bicellular pollen reflects defects in male gametogenesis caused by *bnb1 bnb2 wit1 wit2* quadruple mutations.

Thereafter, we analyzed *in-vitro* germinated pollen tubes. In the WT-*RHT* line, the MGU was found at the apical region of the pollen tube, with the vegetative nucleus ahead of the two sperm nuclei (**Figures 1C, D**). However, the vegetative nuclei of *wit1 wit2* double mutant and *bnb2 wit1 wit2* triple mutant plants were frequently separated from sperm nuclei and located in the base of the pollen tube (**Figures 1C–E**). This phenotype indicates a loss of motive force in the vegetative nucleus. The remaining approximately half of the pollen tubes showed apical transport of MGU in *wit1 wit2* and *bnb2 wit1 wit2* mutants (**Figure 1D**). In this type of apical MGU transport, however, almost all the sperm nuclei preceded the vegetative nucleus (**Figure 1E**). Given that the sperm cells and vegetative nuclei are physically connected in the pollen tubes, we suggested that in the MGUs of these pollen tubes, this connection remains unbroken, resulting in the immotile vegetative nuclei being passively pulled by the sperm cells that proceed ahead of them. Thus, the absence of sperm cells is expected to deprive the motility of the vegetative nucleus in *wit1 wit2* mutant pollen tubes. Consistently, we found that the vegetative nucleus was at the basal region in 97% of the unicellular *bnb1 bnb2 wit1 wit2* quadruple mutant pollen tubes segregated from +/*bnb1 bnb2 wit1 wit2* mutant lines (n = 65, **Figures 1C, D**).

The unicellular *bnb1 bnb2 wit1 wit2* mutant pollen tube is an ideal material to investigate how vegetative nuclei function during pollen tube growth because we can exclude the possible contribution of the sperm cells. The first callose plug formation would block the *de novo* transcript supply from the immotile vegetative nucleus to the growing tip region (**Figure 1C**). An analysis of the length of the pollen tubes at 3 HAG demonstrated that the growth of the unicellular *bnb1 bnb2 wit1 wit2* quadruple mutant pollen tubes was comparable with those of the WT pollen tubes, *wit1 wit2* double mutant pollen tubes, and *bnb2 wit1 wit2* triple mutant pollen tubes (**Figure 1F**). To clarify the ability of pollen tubes to respond to attraction signals from the ovule, we pollinated a WT pistil with a single pollen grain and performed aniline blue staining one day after pollination. Among pollen grains from +/*bnb1 bnb2 wit1 wit2* mutant, we distinguished the unicellular *bnb1 bnb2 wit1 wit2* mutant pollen from *bnb2 wit1 wit2* pollen by its unique teardrop morphology under a microscope (**Figure 1A**, lower-left panel). In the single pollen tube guidance assay, the *bnb1 bnb2 wit1 wit2* pollen with tear-drop morphology showed normal germination rate (42.2%) that was comparable with that of WT-Col-0 pollen tubes (63.6%, *p* = 0.085, compared to *bnb1 bnb2 wit1 wit2*) or *bnb2 wit1 wit2* pollen tubes (59.0%, *p* = 0.120, compared to *bnb1 bnb2 wit1 wit2*). Strikingly, we found that *bnb1 bnb2 wit1 wit2* mutant pollen tubes could reach the ovule despite the nuclear-absent condition (**Figure 1G**), albeit the ovule-insertion rate (9.4%) was significantly lower than those of WT pollen (31.8%) and *bnb2 wit1 wit2* pollen tubes (24.6%) (**Figure 1H**). A long pollen tube journey elucidated the functional limitation of enucleated pollen tubes. However, it was clear that some enucleated pollen tubes obtained the capability to reach the ovule. This finding indicates the robustness of the pollen tube to function without the *de novo* transcript supply from the vegetative nucleus, which supports our previous findings on *wit1 wit2 SC-cal* pollen tubes (Motomura et al., 2021).

## Discussion

It is widely believed that a vegetative nucleus is required for pollen tube growth. However, the pollen tubes of the *bnb1 bnb2 wit1 wit2* quadruple mutant and the *wit1 wit2 SC-cal* mutant could grow and even showed targeting behavior towards the ovule after enucleation by the first callose plug formation (Motomura et al., 2021) (**Figure 1**). Based on these findings and other cumulative evidence, we propose plausible mechanisms responsible for the persistent directional growth capability of pollen tubes.

### Precocious gene expression required for persistent pollen tube growth

**Figure 2:**
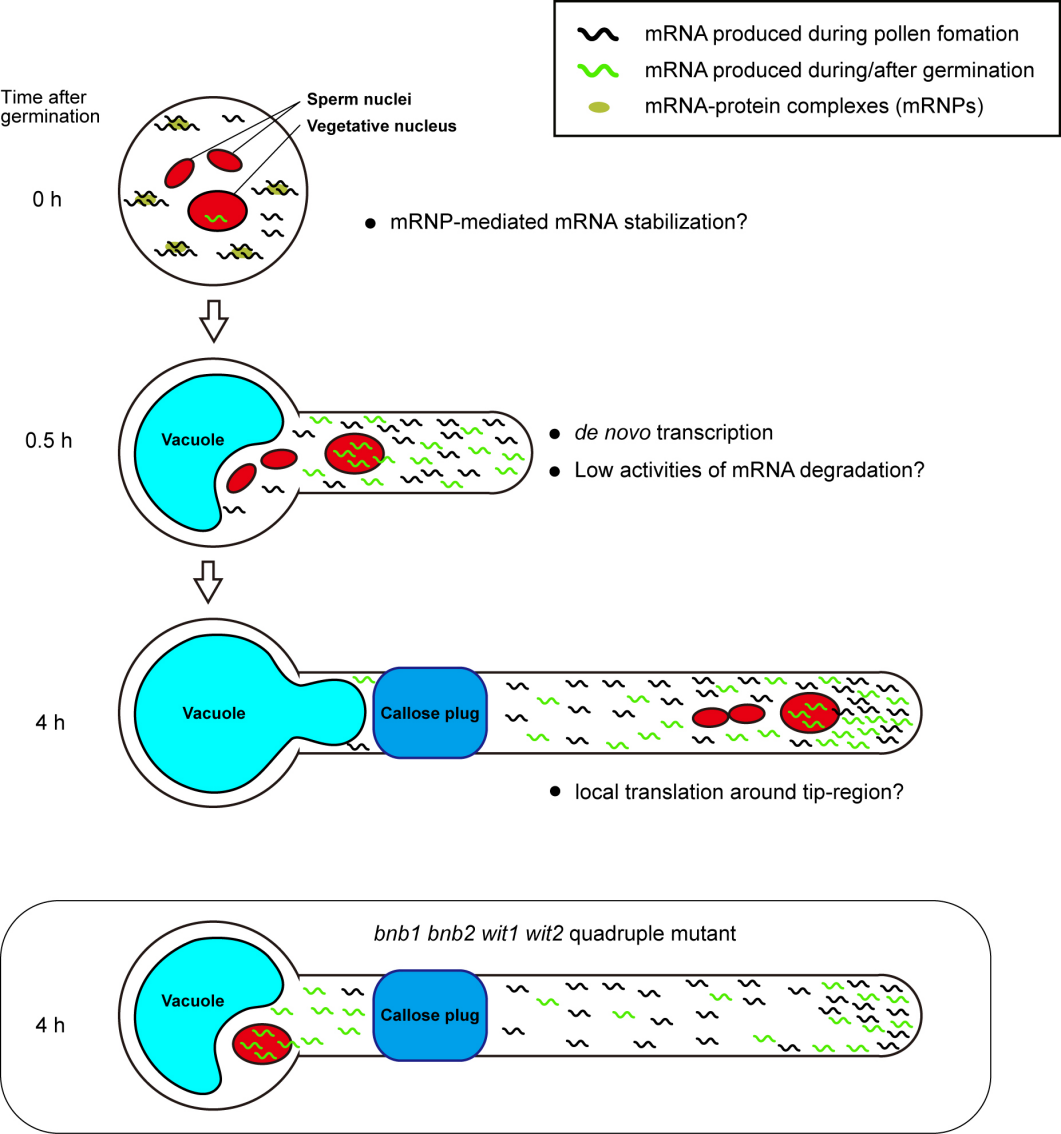
Plausible mechanism for persistent pollen tube growth based on gene expression. Pollen tubes generate many transcripts for their germination and short-time pollen tube growth during pollen tube development. Mature pollen may conserve large amounts of mRNAs in the quiescent mRNP form (0 h). When pollen tubes are ready to germinate, they begin to germinate with the transcription of *de novo* mRNAs within a short period of time. In the germinating pollen tube, reduced mRNA degradation activity may contribute to the accumulation of a large amount of transcripts (0.5 h). In *Arabidopsis* Col-0 accession, the first callose plug is formed at the basal part of the pollen tube within 3 h after germination. Subcellular localization bias of mRNA in pollen tubes is not known; however, local translation at the pollen tube tip may contribute to active pollen tube growth (4 h). In *bnb1 bnb2 wit1 wit2* quadruple mutant pollen tubes, the vegetative nucleus is left at the basal area, implying that *de novo* transcripts are not supplied to the apical region. However, transcripts that accumulate in the apical region by riding the cytoplasm flow prior to the formation of the first callose plug may maintain persistent pollen tube growth (*bnb1 bnb2 wit1 wit2* quadruple mutant).

We assume that early acquisition of persistent pollen tube growth capability depends on a combination of mRNA storage in mature pollen and *de novo* transcription in a short period of time after germination. The mRNAs accumulated in mature pollen are sufficient to induce germination because various species can germinate their pollen tubes on media containing transcription inhibitors, such as actinomycin D (Honys and Twell, 2004; Hao et al., 2005). Pollen grains may have a special mRNA storage system. In *Nicotiana tabacum* and *A. thaliana*, mature pollen contains mRNA-protein complexes (mRNPs) (Honys et al., 2009; Scarpin et al., 2017; Hafidh et al., 2018). These mRNPs resemble stress granules that isolate mRNA from ribosomes, preventing *de novo* protein synthesis in somatic tissues under stress conditions. Recently, Sze et al. (2021) proposed that pollen accumulates transcripts in a quiescent form of mRNP and release the transcripts during rehydration, prior to pollen tube germination (**Figure 2**, 0 h).

Precocious mRNA accumulation alone appears to be insufficient for persistent pollen tube growth. Pollen tubes germinated on a medium containing a transcriptional inhibitor showed growth retardation in various species, including *Tradescantia*, *Lobelia*, *Aeschynanthus*, *Iris*, *Hippeastrum*, *Narcissus*, and *Endymion*, indicating the importance of the transcripts synthesized after pollen tube germination (Mascarenhas, 1975). In contrast, the pollen tubes of the +/*bnb1 bnb2 wit1 wit2* quadruple mutant and *wit1 wit2 SC-cal* mutant showed normal pollen tube growth without any retardation (Motomura et al., 2021) (**Figure 1**). The difference between these two observations may be because of the presence of *de novo* transcripts supplied immediately after germination. The pollen tubes of the *bnb1 bnb2 wit1 wit2* quadruple mutant and *wit1 wit2 SC-cal* mutant have vegetative nuclei at the basal region; thus, these pollen tubes can transport *de novo* transcripts into their apical growing region (**Figure 2**, 0.5 h). This mRNA transport continues until the first callose plug formation, which occurs approximately a few hours after germination. In transcriptome analyses, pollen tubes growing through a style 4 h after pollination show a very different gene expression pattern from that of mature pollen grains (Qin et al., 2009; Chen et al., 2014). Although the functions of most of the induced genes remain unknown, an R2R3-type MYB transcription factor, MYB120, starts to accumulate after pollen tube germination and regulates pollen tube discharge in the ovules with its paralogs MYB97 and MYB101 redundantly (Leydon et al., 2013). These reports indicate that a subset of genes expressed after germination is required for the functional maturation of pollen tubes. We anticipate that these genes play key roles in the persistent directional growth capability of pollen tubes.

### Stability and transport of transcripts in pollen tubes

In addition to the production of sufficient transcripts, mRNA stability would also be important for robust pollen tube growth after enucleation. The pollen tubes may be able to recycle mRNA molecules and continue to use them for long periods compared to somatic cells (**Figure 2**). RNA exosomes or decapping proteins are key enzymes of RNA metabolism that are ubiquitously expressed during the transition of cell identity and function through RNA degradation. Mutants of these factors show severe developmental defects during gametogenesis and embryogenesis (Motomura et al., 2012; Kumakura et al., 2013; Motomura et al., 2020). Pollen tube growth also accompanies drastic changes in the gene expression profile and morphology; however, no phenotypes have been reported in these mutant pollen tubes. Moreover, gene sets with an ontology term RNA metabolic process are less expressed in pollen tubes (Poidevin et al., 2021). These findings support the low RNA metabolic activity in the pollen tubes (**Figure 2**, 0.5 h).

Not only the suppression of mRNA degradation but also the directional mRNA transport and local activation of protein synthesis may be the key mechanisms responsible for the robustness of pollen tube growth. Axon guidance in animal neurons is often compared to pollen tube growth because of similarities in the active tip growth and the response behavior against directional cues. Neurons produce mRNA in the nucleus at the cell body and then transport them to the growth cone (Rodriguez et al., 2008). During axonal mRNA transport, mRNAs form RNPs and are most likely to repress translation. In contrast, mRNAs become translationally active at the growth cone. Interestingly, abnormal neurite outgrowth was observed when local translation was impaired, indicating the importance of axonal mRNA transport and local protein synthesis in neurons (Rodriguez et al., 2008). The active transport of specific mRNA and local protein synthesis in the pollen tube have not been reported so far; however, a recent study has uncovered a regulatory mechanism of local protein synthesis in *A. thaliana* root hairs, which are tip-growing cells (Zhu et al., 2020). These studies improve the understanding of the biology of RNA binding proteins in the pollen tube and enable the exploration of possible mechanistic links to the animal axon guidance (Billey et al., 2021; Duarte-Conde et al., 2022) (**Figure 2**, 4 h).

### Future directions for the study of pollen tube capability

The duration of pollen tube growth varies in different organisms and is sometimes more than 24 h (O’Neill, 1997; Borgardt and Nixon, 2003). Precocious mRNA production as well as stabilization of mRNA will provide unusual and persistent growth capabilities to pollen tubes. The mechanisms of mRNA transport may also have positive effects on pollen tube germination by saving transcription time and facilitating germination and tip growth, thereby providing an opportunity to win a competitive race among pollen tubes and increase reproductive fitness (Erbar, 2003). Moreover, mRNA reservoirs in pollen tubes possibly contribute toward the quick response against biotic and/or abiotic environmental stresses, as the expression of genes involved in DNA repair and defense response is upregulated in pollen tubes growing through a style (Qin et al., 2009). In the context of pollen biology, enucleated pollen tubes from the +/*bnb1 bnb2 wit1 wit2* mutant are valuable for analyzing the persistent growth of pollen tubes and illuminating the functional limitation of the early set of transcripts.

## Data availability statement

The original contributions presented in the study are included in the article/supplementary material. Further inquiries can be directed to the corresponding authors.

## Author contributions

KM and DM designed the study, conducted the experiments, analyzed pollen tube growth, and drafted the manuscript. NS analyzed the pollen phenotypes of the mutants. AT and SY provided critical advice and reviewed the manuscript. All authors have contributed to the manuscript and approved the submitted version.

## Funding

This work was supported by JSPS KAKENHI (Grant nos. JP19H04869, JP20H03280, JP20K21432, JP20H05422, JP20H05778, and JP20H05781 awarded to DM; and Grant nos. JP19K23759, JP20K15822, and JP22K15147 awarded to KM; JP20H05780 awarded to SY); Asahi Glass Foundation (Research Grant to SY); JST PRESTO (Grant no. JPMJPR20D9 awarded to KM); Yokohama City University (Academic Research Grant to DM; Development Fund to DM; and Strategic Research Promotion Grant no. SK1903 to DM); Japan Science Society (Sasakawa Scientific Research Grant to KM); Takeda Science Foundation (research Grant to KM); and Ritsumeikan Global Innovation Research Organization, Ritsumeikan University (Third-Phase R-GIRO Research Grant to KM and AT).

## Acknowledgments

We thank K. Tamura and I. Mayer for providing the *wit1 wit2* mutant; D. Kurihara for providing the *pRPS5A:H2B-tdTomato*; K. Matsuura-Tokita for performing the *in-vitro* germination assay; T. Kinosita for various support; and M. Tsukatani, H. Ikeda, H. Kakizaki, A. Matsumoto, A. Saito, and R. Shibayama for their technical support. We would like to thank Editage (www.editage.com) for English language editing.

## Conflict of interest

The authors declare that the research was conducted in the absence of any commercial or financial relationships that could be construed as a potential conflict of interest.

## Publisher’s note

All claims expressed in this article are solely those of the authors and do not necessarily represent those of their affiliated organizations, or those of the publisher, the editors and the reviewers. Any product that may be evaluated in this article, or claim that may be made by its manufacturer, is not guaranteed or endorsed by the publisher.
